# Upper Gastrointestinal Cancer Surveillance in Lynch Syndrome

**DOI:** 10.3390/cancers14041000

**Published:** 2022-02-16

**Authors:** Shria Kumar, Natalie Farha, Carol A. Burke, Bryson W. Katona

**Affiliations:** 1 Division of Digestive Health and Liver Diseases, University of Miami Miller School of Medicine, Miami, FL 33136, USA; shriakumar@med.miami.edu; 2 Department of Internal Medicine, Cleveland Clinic, Cleveland, OH 44195, USA; farhan@ccf.org; 3 Department of Gastroenterology, Hepatology & Nutrition, Cleveland Clinic, Cleveland, OH 44195, USA; burkec1@ccf.org; 4 Division of Gastroenterology and Hepatology, Perelman School of Medicine, University of Pennsylvania, Philadelphia, PA 19104, USA

**Keywords:** Lynch syndrome, upper gastrointestinal cancer, surveillance, upper endoscopy

## Abstract

**Simple Summary:**

Lynch syndrome is the most common cause of hereditary colorectal cancer, but is also associated with increased extracolonic cancer risk, including upper gastrointestinal cancers. While there is agreement regarding the benefit of frequent colonoscopic surveillance in Lynch syndrome, there remains a lack of consensus on the use of upper gastrointestinal cancer surveillance. Here, we review the upper gastrointestinal cancer risks in Lynch syndrome, the varying guideline recommendations in this area, and the published outcomes of upper gastrointestinal cancer surveillance in this high-risk population. Finally, we highlight ongoing controversies in upper gastrointestinal cancer surveillance and opine on how upper gastrointestinal cancer surveillance can be incorporated into a Lynch syndrome risk management program. Upper gastrointestinal cancer surveillance is an increasingly studied area of risk management in Lynch syndrome, and continued research will be vital in determining how to best incorporate this surveillance in these high-risk patients.

**Abstract:**

Lynch syndrome is a common hereditary cancer predisposition syndrome associated with increased digestive cancer risk including colorectal, gastric, and duodenal cancers. While colorectal cancer surveillance is widely accepted to be an important part of a comprehensive Lynch syndrome risk management plan, the use of upper gastrointestinal cancer surveillance in Lynch syndrome remains more controversial. Currently, upper gastrointestinal cancer surveillance guidelines for Lynch syndrome vary widely, and there is no consensus on who should undergo upper gastrointestinal cancer surveillance, how surveillance should be performed, the age at which to initiate surveillance, or how often individuals with Lynch syndrome should undergo upper gastrointestinal cancer surveillance. Fortunately, research groups around the world have been focusing on upper gastrointestinal cancer surveillance in Lynch syndrome, and recent evidence in this field has demonstrated that upper gastrointestinal cancer surveillance can be performed with identification of precancerous lesions as well as early-stage upper gastrointestinal cancers. In this manuscript, we review the upper gastrointestinal cancer risks in Lynch syndrome, differing guideline recommendations for surveillance, outcomes of upper gastrointestinal cancer surveillance, and controversies in the field, and we provide a framework based on our collective experience with which to incorporate upper gastrointestinal cancer surveillance into a risk management program for individuals with Lynch syndrome.

## 1. Introduction

Lynch syndrome is among the most common hereditary cancer predisposition syndromes, leading to a substantially increased risk of colorectal cancer (CRC) [1]. The risk for extra-colonic cancers is also increased, including endometrial and ovarian cancers, as well as gastric, biliary, urinary tract, pancreatic, skin, and small bowel cancers [2]. Lynch syndrome results from germline pathogenic variants in genes involved in the DNA mismatch repair pathway including *MLH1, MSH2, MSH6, PMS2* as well as *EPCAM*, whose deletion leads to hypermethylation and silencing of *MSH2* [2,3,4]. Given the role of Lynch syndrome-related genes in mismatch repair, Lynch syndrome-associated tumors classically exhibit mismatch repair deficiency associated with microsatellite instability and increased tumor mutational burden [2,3,4,5].

The lifetime cancer risk for some patients affected with Lynch syndrome can be over 50% for colorectal and endometrial cancer [6]. There is consensus among guidelines recommending aggressive colorectal surveillance to improve mortality from CRC, as well as prophylactic hysterectomy to reduce endometrial cancer risk [7,8]. The role for surveillance of other Lynch syndrome-related cancers with lower, but still substantially elevated, risk compared to the general population remains an area of persistent uncertainty. This is particularly true for cancers of the upper gastrointestinal (UGI) tract. While the general population has a lifetime risk of 0.9% and 0.3% for gastric cancer and duodenal cancer, respectively, in Lynch syndrome, this risk can be as high as 9% and 11%, respectively [7,9,10,11,12]. However, there are marked variations of cancer risk by age and by gene. For example, the Prospective Lynch Syndrome Database demonstrated marked differences in UGI cancer risk by age and pathogenic variant, with UGI cancers occurring less frequently among younger age groups [13]. Numerous guidelines both within and outside the United States address UGI surveillance in Lynch syndrome, but there remains a lack of consensus regarding the efficacy of UGI surveillance and the surveillance approach (Table 1). When UGI surveillance is performed in Lynch syndrome, it is typically carried out with upper endoscopy or esophagogastroduodenoscopy (EGD), which is a diagnostic endoscopic procedure that visualizes the UGI tract. In addition to inspection, biopsies obtained during upper endoscopy can evaluate for *Helicobacter pylori* infection and precancerous lesions, such as atrophic gastritis, intestinal metaplasia, and dysplasia. Similar to colonoscopy, upper endoscopy can be repeated at regular intervals to surveil for cancers, including those in the esophagus, stomach, and duodenum.

The multitude of guidelines addressing UGI surveillance in Lynch syndrome are not uniform and vary in multiple aspects (Table 1). While some guidelines do not recommend routine surveillance and instead recommend only evaluating for *Helicobacter pylori* infection [14], newer guidelines often recommend surveillance of higher risk groups, yet the definition of a high-risk group is not consistent [7,15,16,17]. The recommended age of initiation of UGI surveillance also varies greatly, with some guidelines not proposing a specific age to initiate surveillance [15,16], while others recommend initiation at different times between ages 30–40 years [7,17,18,19]. Lastly, the suggested surveillance interval is also inconsistent, with recommended intervals ranging between every 1 to 5 years depending on the particular guideline [7,15,16,17,18,19].

The lack of consensus regarding support of UGI surveillance in Lynch syndrome is related to the lack of evidence supporting its utility. However, over the last several years there have been a plethora of studies providing important data highlighting the role of UGI surveillance in Lynch syndrome, strengthening the evidence supporting its regular use as part of a comprehensive risk-management program for Lynch syndrome. Herein, we review the literature regarding UGI cancer risk and outcomes of endoscopic surveillance of UGI cancers in Lynch syndrome, describing areas of controversy, and offering our own perspectives on how to effectively incorporate UGI surveillance into the care of patients with Lynch syndrome.

## 2. Esophageal Cancer

Esophageal cancer is not classically considered a Lynch syndrome-associated malignancy. Classical risk factors for esophageal adenocarcinoma include male sex, increasing age, obesity, chronic acid reflux, and Barrett’s esophagus [22]. While the incidence of esophageal adenocarcinoma is increasing in western countries [23,24], worldwide, squamous cell cancer remains the more common esophageal cancer and is associated with smoking and alcohol intake [22]. These risk factors can co-exist in individuals with Lynch syndrome, and may predispose these patients to esophageal cancer, despite esophageal cancer being outside of the classic spectrum of Lynch syndrome-associated UGI cancers.

Endoscopy can detect esophageal cancer, even those at an early stage [25]. Using a combination of high-definition white light, narrow band imaging, and both targeted and random biopsy, endoscopy can detect mucosal abnormalities that indicate preneoplastic or neoplastic conditions of the esophagus [26,27,28,29]. These findings can include nodularity, altered pit pattern or vascularity, depression, and protruding lesions, with upper endoscopy being recommended for esophageal cancer surveillance for individuals at high risk [30]. Accordingly, there are guidelines for screening of esophageal cancer in those at high risk, which in western countries focus primarily on surveilling for lesions associated with Barrett’s esophagus [26,27,28,29].

Unlike gastric and duodenal findings, there is a paucity of data about esophageal findings in patients with Lynch syndrome. A recent study of 323 individuals with Lynch syndrome undergoing EGD surveillance identified 6.5% with Barrett’s esophagus; of those with Barrett’s esophagus, 9.5% had dysplasia and 1 patient (0.3% of the overall cohort) had Barrett’s esophagus-related esophageal adenocarcinoma [31]. In this study, the relatively frequent finding of Barrett’s esophagus was twice the prevalence reported by the only other study of EGD surveillance in Lynch syndrome to report Barrett’s esophagus, in which of 217 patients, 7 (3.2%) were identified to have Barrett’s esophagus, with only 1 of these 7 patients having dysplasia (authors’ own unpublished data) [32]. The prevalence of Barrett’s esophagus in the general population is unknown—with estimates from clinical and modeling studies ranging from ~1% to above 6% [33,34,35]. With uncertain estimates within Lynch syndrome, it is unclear if Lynch syndrome portends a higher risk of Barrett’s esophagus than the general population.

Mismatch repair-deficient esophageal adenocarcinomas have also been identified in Lynch syndrome. A recent report identified a stage I esophageal adenocarcinoma in a 56-year old female *MSH2* carrier with known Barrett’s esophagus who was undergoing endoscopic surveillance [31,36]. Additionally, two case reports including one of an 84-year old woman and the other a 35-year old man, both with *MSH2*-associated Lynch syndrome, described esophageal adenocarcinoma, both lacking expression of *MSH2* on immunohistochemistry (IHC) [37,38]. Among these two lesions, one was in a pedunculated polyp, while the other was in a cervical inlet patch. The cervical inlet patch, with underlying heterotopic gastric mucosa, is a common and typically incidental finding with minimal, if any, clinical relevance [39]. The risk of adenocarcinoma arising from heterotopic gastric mucosa is thought to be low [39,40], but cervical inlet patches should be carefully examined in all individuals undergoing upper endoscopy. While these reported cases of esophageal adenocarcinoma in Lynch syndrome were mismatch repair deficient, the majority of mismatch-deficient esophageal adenocarcinomas are not related to Lynch syndrome, and 3–5% of esophageal adenocarcinomas have been found to harbor somatic mismatch repair deficiency [41,42].

In summary, esophageal cancer is not recognized as a classic Lynch syndrome-associated malignancy. However, given that upper endoscopy can detect esophageal cancer and its precursor lesions, endoscopic surveillance for UGI malignancies in Lynch syndrome may also detect esophageal neoplasia. For example, in a series of individuals with Lynch syndrome patients undergoing UGI surveillance, an esophageal squamous cell cancer was identified, highlighting the importance of careful endoscopic examination of the esophagus in Lynch syndrome, even for non-classical neoplastic associations [32].

## 3. Gastric Cancer

Individuals with Lynch syndrome are at increased risk of gastric cancer [1,43]. Gastric cancer risk varies by pathogenic variant with *MLH1* and *MSH2* carriers having a higher risk compared to *MSH6* and *PMS2* carriers [44]. A recent summary from the National Comprehensive Cancer Network (NCCN) highlights the range of gene-specific gastric cancer risk in Lynch syndrome (Table 2) [7]. This summary demonstrated lifetime risk was 5–7% for *MLH1* carriers, 0.2–9% for *MSH2* and *EPCAM* carriers, and <1–7.9% in *MSH6* carriers, and importantly notes that the risk for *PMS2* carriers is unknown, illustrating the uncertainty of whether gastric cancer risk is increased for *PMS2* carriers.

Multiple guidelines recommend surveillance based on patient-specific risk factors. While risk factors for Lynch syndrome-related gastric cancers have been identified and include male sex, increasing age, and family history of gastric cancer [44], gastric cancer has also been diagnosed among patients without these risk factors [32,45,46,47]. Furthermore, guidelines are not consistent in their definition of who is considered a high-risk individual. For example, the NCCN recognizes risk factors that include male sex, older age, *MLH1* or *MSH2* pathogenic variant, first-degree relative with gastric cancer, Asian ethnicity, residing or immigrating from countries with high incidence of gastric cancer, and a history of chronic autoimmune gastritis, gastric intestinal metaplasia, or gastric adenomas [7], but the American College of Gastroenterology (ACG) recommends risk stratification based on family history alone [18]. As such, it remains uncertain how to best utilize risk factors to determine which patients with Lynch syndrome should undergo UGI surveillance. It is also likely that UGI surveillance should be dependent on the particular Lynch syndrome gene affected as there are multiple studies demonstrating differential risk by pathogenic variant, including a study among individuals testing positive for Lynch syndrome at a commercial laboratory in the United States [44], the international, multicenter Prospective Lynch Syndrome Database [48], and studies from European cancer registries [49,50,51].

The endoscopic appearance of gastric cancers in Lynch syndrome has been described by several studies. In a series of 217 patients with Lynch syndrome undergoing upper endoscopic surveillance, gastric cancer was detected as a visible mass or a polyp in six patients (2.8%) [32]. In this series, pre-cancerous upper endoscopy findings identified included gastric intestinal metaplasia (18, 8.3%) and *Helicobacter pylori* (6, 2.8%). Gastric neoplasia has been reported in two additional series of UGI Lynch syndrome surveillance. In 323 asymptomatic patients undergoing EGD surveillance, two patients (0.6%), both with an *MSH2* pathogenic variant, were found to have gastric cancer—one type 3 gastric neuroendocrine tumor and one gastric adenocarcinoma identified as a solitary ulcerated mass in the proximal stomach [31]. Notably, both gastric cancers were identified at an early stage. In this series, 32 patients (6%) had at least one lesion associated with gastric carcinogenesis, including 4% with *Helicobacter pylori*, 6% with gastric intestinal metaplasia, 2% with gastric hyperplastic polyps > 5mm, and 1% with gastric adenomas. In another study of 247 asymptomatic patients with either a mismatch repair pathogenic variant or clinical features suggestive of Lynch syndrome undergoing EGD surveillance over a mean of 5.7 years, an average of 3.5 EGDs were conducted at an interval of 2.3 years between exams [45]. Gastric cancer was detected in two patients (0.8%), one early stage; antral cancer was detected in the setting of *Helicobacter pylori* at first EGD in an *MSH6* carrier and the other in the cardia at late stage 5 years after a prior EGD in an *MSH2* carrier. Pre-cancerous lesions of the stomach and duodenum were described in eight (3.2%) patients. The gastric findings included gastric foveolar dysplasia, gastric adenomas, and intestinal metaplasia in 10.1%, and chronic gastritis with *Helicobacter pylori* in 6.9%.

Despite these studies describing gastric cancers in Lynch syndrome, the pathogenesis of gastric cancer in Lynch syndrome remains incompletely understood. In sporadic cases, gastric carcinogenesis proceeds down a defined pathway from chronic gastritis to gastric adenocarcinoma (the “Correa cascade”): chronic gastritis, atrophic gastritis, intestinal metaplasia, dysplasia, then finally, adenocarcinoma [52]. Infection with *Helicobacter pylori* is thought to be a necessary component of this cascade [53]. While consensus guidelines frequently recommend testing for and eradication of *Helicobacter pylori* infection, the rates of *Helicobacter pylori* infection in individuals with Lynch syndrome appear to vary, along with the precancerous lesions associated with sporadic gastric carcinogenesis. Among a cohort of 255 individuals with Lynch syndrome, seven patients (2.7%) developed gastric cancer, including five with concomitant chronic immune gastritis [54]. On the other hand, the frequency of intestinal metaplasia did not differ between patients who developed cancer versus those who did not and in the overall cohort, *Helicobacter pylori* infection itself was rare (2.8%) [32]. Other studies, including one from the Dutch Hereditary Cancer Registry, found that *Helicobacter pylori* was not associated with increased gastric cancer risk in Lynch syndrome [49,55]. A French study also found no association between preceding histologic abnormalities and future gastric cancer risk [47]. Despite this, all published guidelines routinely recommend testing for and eradicating *Helicobacter pylori* infection—though they do not uniformly recommend biopsies for histological evaluation [15,16,18,19,56]. While *Helicobacter pylori* infection is considered a class I carcinogen and eradication decreases future gastric cancer risk, it is not clear that *Helicobacter pylori* infection is driving the majority of Lynch-associated gastric cancers [57].

Another intriguing theory about Lynch syndrome-associated gastric carcinogenesis is whether these cancers develop via an accelerated alternative pathway. In Lynch syndrome-associated CRC, there is a well-recognized accelerated adenoma to carcinoma progression, where malignant transformation occurs within a few years, as opposed to 10 or more years in individuals without Lynch syndrome [58]. Accordingly, colonoscopic surveillance for Lynch syndrome is recommended at short intervals [7,15,17,18]. While the same pathway is not well-established for gastric carcinogenesis, a 2018 German study of 44 subjects undergoing surveillance noted the possibility of endoscopic surveillance being able to detect and remove adenomas at a precancerous stage, but in this study, adenomas were rare (only five detected adenomas) [59]. However, the ability to detect early-stage gastric cancers by endoscopic surveillance has been better established. In a 2020 study of 217 patients, 80% of detected cancers were stage I [32]. In a 2021 study of 323 patients, all gastric cancers were detected at early stage [31]. In another 2021 study of 1128 individuals, patients who were found to have gastric cancer while undergoing regular surveillance were diagnosed significantly more often in early-stage disease than those gastric cancers detected through work-up of symptoms (83% vs. 25%; *p* = 0.02) [46]. In all of these studies, gastric cancers that developed during surveillance were typically detected within 2 years of a prior surveillance exam, thus further supporting the possibility of an accelerated carcinogenesis pathway in Lynch syndrome-associated gastric cancers. Notably, studies have not uniformly demonstrated a clear survival benefit resulting from UGI endoscopic surveillance, and this, along with assessing the cost-effectiveness of an UGI surveillance program, is an essential next step in the field.

Finally, gastric neuroendocrine tumors in Lynch syndrome have not been consistently reported; however, a study of 323 patients with Lynch syndrome underdoing endoscopic surveillance did detect one, with loss of *MSH2* protein expression [31]. As more UGI surveillance data are collected on Lynch syndrome, this may be an area of further study.

## 4. Duodenal Cancer

Duodenal cancers are rare, occurring in only 0.3% of the general population [10,12]. Individuals with Lynch syndrome are at higher risk of duodenal cancer with a relative risk of 30 to 100 times that of the general population. As with gastric cancer the risk of duodenal cancer is dependent on pathogenic variant (Table 2). While for those with a *MLH1* or *MSH2* pathogenic variant the lifetime risk of duodenal cancer is as high as 10–11%, the risk for carriers of other pathogenic variants is widely ranging, with suggestion that *PMS2* carriers have a risk no higher than the general population [10,12].

Few studies have focused on duodenal cancers in Lynch syndrome; however, similar to other digestive Lynch syndrome-related cancers there have been histologic and molecular findings of microsatellite instability in duodenal cancers [60,61]. In a French cohort of 154 individuals with Lynch syndrome undergoing endoscopic evaluation, three duodenal cancers were noted, all in *MSH2* carriers [62]. These cancers appeared as flat lesions in the first or second portion of the duodenum. Among three US cohort studies including a total of 825 individuals with Lynch syndrome, eight patients developed duodenal cancer [26,27,39]. Seven were duodenal adenocarcinomas and one was a neuroendocrine tumor. Three of the patients had variants in *MSH2*, three in *MLH1*, one in *PMS2* and the neuroendocrine tumor was identified in a patient who met revised Bethesda criteria. Current data support that carriers of a *MSH2* or *MLH1* pathogenic variant have the highest duodenal cancer risk amongst all individuals with Lynch syndrome [50,51]. Whether small bowel neuroendocrine tumors are associated with Lynch syndrome is not established as only a few cases have been reported [36,45]. Of the published reports regarding GI neuroendocrine tumors in Lynch syndrome, two have been in the colon [63], one in the rectum [63], and three in the small bowel [64].

A recent German study evaluating the detection of duodenal cancer in Lynch syndrome identified 49 duodenal cancers in 47 patients, with the majority (91%) of patients having a *MLH1* or *MSH2* pathogenic variant [65]. While the median age at diagnosis of duodenal cancer was 51.7 years, 10% of patients were less than 35 years old at the time of diagnosis. Patients under EGD surveillance had earlier-stage (stage I–IIA) detection in 77% versus 29% of patients in the non-surveillance group. Duodenal adenomas were reported including one diagnosed synchronous with a duodenal cancer and three detected five, six, and twelve months before a duodenal cancer diagnosis. This study did not report if the adenomas were completely resected as incompletely resected duodenal adenomas may have been the precursor to the duodenal cancers detected. In this study, those in the surveillance group underwent an average of 8.1 upper endoscopies per patient (and 11.3 colonoscopies per patient). Those cancers detected outside surveillance were in patients who underwent 4.9 endoscopies per patient (and 6.8 colonoscopies per patient), suggesting some difference in adherence, but also that surveillance requires more procedures.

The precursors of duodenal cancer in Lynch syndrome are presumed to be duodenal adenomas; however, most studies have not reported a high rate of duodenal adenoma identification. In recent studies from Western populations with Lynch syndrome, duodenal adenomas have been reported in 1.5–2.4% of the cohorts [31,32,44,62]. Whether mismatch repair-deficient crypts may also serve as duodenal adenocarcinoma precursors in Lynch syndrome, similar to colonic mismatch repair deficient crypts, is currently unknown. Additionally, little is known about the risk factors for Lynch syndrome-associated duodenal cancers [66,67]. Future studies should evaluate identifiable risk factors for duodenal neoplasia to better risk stratify patients for surveillance, determine whether surveillance prevents death from duodenal cancers, and assess the cost-effectiveness of UGI surveillance.

## 5. Current Guidelines

Guidelines vary markedly with recommendations for UGI surveillance in Lynch syndrome. As is evident from Table 1, there is a lack of consensus on: (1) whether or not to perform an initial UGI cancer surveillance exam or follow up surveillance (2) the age to initiate surveillance and (3) the interval to perform surveillance. *Helicobacter pylori* testing and eradication is universally endorsed; however, universal assessment for premalignant conditions associated with gastric cancer such as atrophic gastritis and intestinal metaplasia remains a point where there is lack of consensus.

## 6. UGI Surveillance Implementation and Controversies

Given the increased risk of UGI cancers in Lynch syndrome and the current data highlighting early-stage detection of UGI cancers in Lynch syndrome (Table 3), we suggest that surveillance for UGI cancers in Lynch syndrome should be a part of a routine Lynch syndrome risk management plan. Furthermore, we would recommend that all individuals with Lynch syndrome who undergo UGI surveillance do so in centers with Lynch syndrome expertise, which will help facilitate prospective data collection on UGI surveillance outcomes.

**Figure 1:**
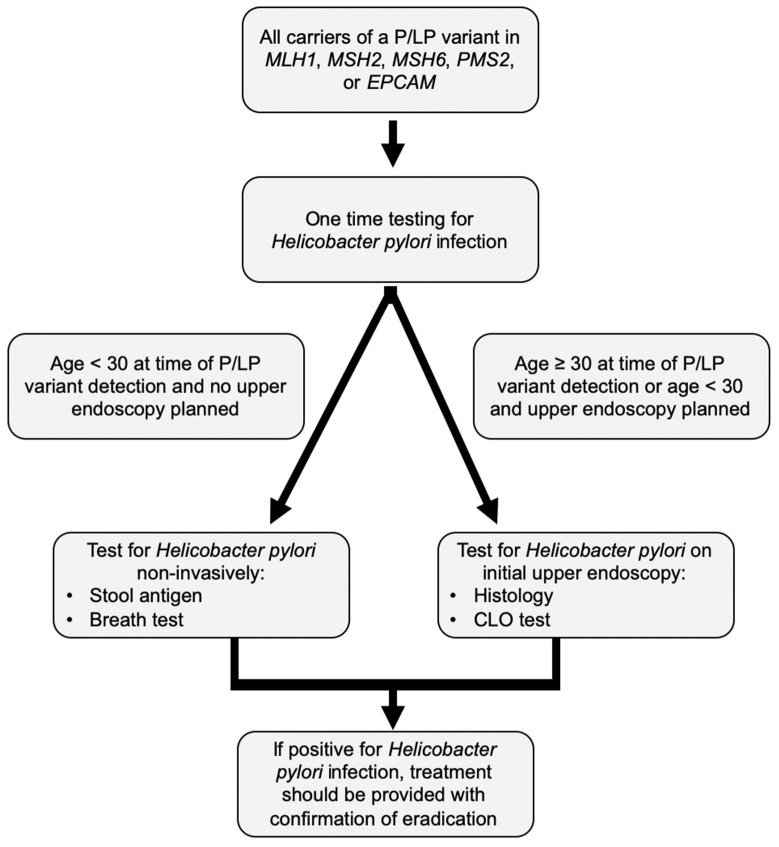
Authors’ approach to *Helicobacter pylori* screening in Lynch syndrome. P/LP variant: Pathogenic/Likely pathogenic variant; CLO test: Campylobacter-like organism test (also known as the Rapid Urease Test).

Based on our collective experience, we would recommend that at the time of Lynch syndrome diagnosis, testing for *Helicobacter pylori* should be undertaken, with treatment of a positive result and confirmation of *Helicobacter pylori* eradication (Figure 1). For those under age 30, non-invasive testing can be performed, whereas for those age 30 and older, testing can be performed at the time of surveillance upper endoscopy. We suggest random biopsies from the gastric antrum and body be obtained on the initial upper GI exam, and on subsequent exams based on suspicious visual findings to allow for detection of gastric precursor lesions, which if present may necessitate shorter surveillance intervals. Upper endoscopy should also be performed at the time the individual is undergoing surveillance colonoscopy, rather than as a standalone procedure, which minimizes additional anesthesia and adds little risk or procedure time for patients already undergoing colonoscopy [7,68,69]. Additionally, push enteroscopy can be considered instead of routine upper endoscopy to allow for assessment of the complete duodenum and proximal jejunum, though there is no clear data to support this.

**Figure 2:**
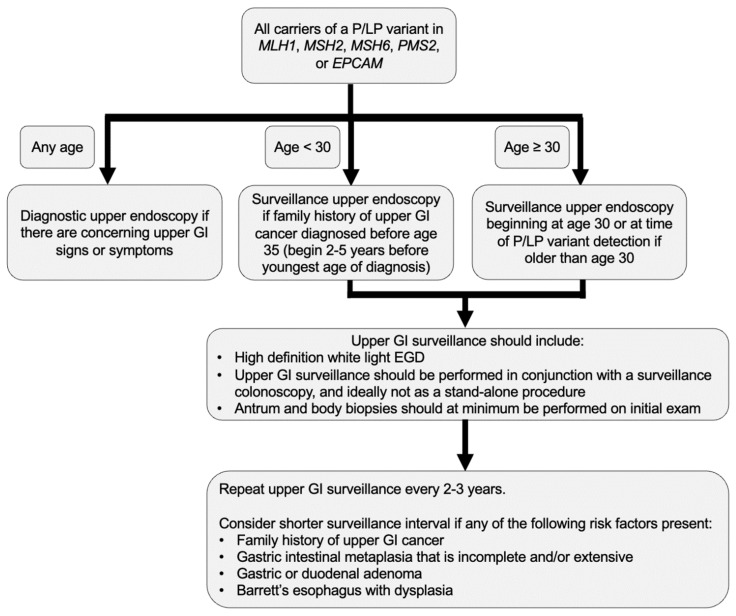
Authors’ approach to Lynch syndrome upper GI cancer surveillance. P/LP variant: Pathogenic/Likely pathogenic variant; GI: Gastrointestinal; EGD: Esophagogastroduodenoscopy.

It is known that colorectal and gynecologic-related cancers often develop at earlier ages in Lynch syndrome compared to the general population [7,70], and data confirm this in UGI cancers as well [49,54,65]. Therefore, we advocate for early initiation of UGI surveillance in Lynch syndrome beginning at age 30, or 2–5 years before the youngest UGI cancer in the family, whichever is earliest (Figure 2). While this surveillance should ideally be performed simultaneously with a surveillance colonoscopy, expeditious diagnostic upper endoscopy is warranted to evaluate concerning UGI signs or symptoms.

The frequency of surveillance remains unknown, yet data from multiple studies suggest consideration of a short UGI surveillance interval [32,44,59]. We recommend a 2–3-year surveillance interval with consideration of shorter surveillance intervals if there are any risk factors, such as family history of UGI cancer, Barrett’s esophagus with dysplasia, gastric intestinal metaplasia that is incomplete and/or extensive, or duodenal or gastric adenomas. Random biopsies from the gastric antrum and body should at minimum be obtained on the initial exam to allow for detection of *Helicobacter pylori* as well as gastric precursor lesions including gastric intestinal metaplasia, which if present may necessitate shorter surveillance intervals (Figure 2).

Of note, data are lacking regarding the association between *Helicobacter pylori* and gastric cancer in Lynch syndrome, as well as family history; therefore, the above is based on our collective centers’ experience. Most data suggest that *MSH2* and *MLH1* pathogenic variants are associated with an increased risk of UGI cancer, but we believe that at this time there are not enough data available to stratify surveillance based on pathogenic variant alone. As we note above, even patients with “lower risk” Lynch syndrome variants have had UGI cancer and neoplasia detected on surveillance. Furthermore, it is possible with additional studies of UGI surveillance in Lynch syndrome that gene-specific recommendations may be appropriate; however, until those data are available, we recommend surveillance among all pathogenic variants.

The duodenum is the most frequent site of small intestinal cancers [71]. The duodenum can be evaluated through the third portion with standard EGD, and can be evaluated in its entirety with push enteroscopy. However, push enteroscopy poses additional procedural time and therefore slightly increased risk, and its benefit in detection of early lesions remains unknown. Future studies should compare the effectiveness and burden of push enteroscopy versus standard upper endoscopy in Lynch syndrome.

Overall, the topic of upper endoscopic surveillance in Lynch syndrome is not without its controversies, which include the age to initiate surveillance, the frequency of surveillance, and whether UGI surveillance should be targeted to certain Lynch syndrome sub-groups. Future studies will be needed to clarify these areas, particularly whether UGI surveillance is cost-effective and whether it prevents death from UGI cancer in Lynch syndrome. Large prospective consortium studies may be best suited to effectively answer these questions, given the relatively small numbers of single-center studies. An important consideration after consensus is reached regarding the need and methods of surveillance is whether there should be quality metrics regarding upper endoscopy for cancer detection in Lynch syndrome, akin to those in colonoscopy. This would ensure that upper endoscopy is of standardized quality across centers.

## 7. Limitations of the Lynch Syndrome UGI Surveillance Literature

It is important to note that the existing Lynch syndrome UGI surveillance literature does have weaknesses, which are important to consider when interpreting the data. First, all of the published studies in this area were performed in a retrospective manner, and therefore may have been subject to bias, including selection bias. Furthermore, the published studies do not assess the impact of UGI surveillance on preventing death from UGI cancers, which is arguably the most important endpoint. A randomized controlled trial of UGI surveillance in Lynch syndrome would be the best method to obtain unbiased data about the efficacy of UGI surveillance in Lynch syndrome; however, it is unlikely that such a trial will ever be performed as currently it would be impractical to randomize individuals with Lynch syndrome to a no-surveillance arm. Instead, large prospective consortium studies of UGI surveillance in Lynch syndrome are likely going to be most effective at generating better data on upper GI surveillance in Lynch syndrome. Another major limitation of this literature is the lack of geographic and racial/ethnic diversity present in the published studies, which have primarily come from Europe and large academic centers in the United States. The outcomes of upper GI surveillance in Lynch syndrome amongst different populations remain unknown, and this certainly also merits further investigation.

## 8. Conclusions

Guidelines regarding surveillance for UGI cancers in Lynch syndrome remain an obfuscated area, posing a source of confusion to patients and providers alike. Recent studies have demonstrated the efficacy of endoscopic surveillance in detecting UGI cancers in patients at an early stage compared to those presenting for diagnostic upper endoscopy due to symptoms. Upper endoscopy is a safe and low burden procedure when coupled with colonoscopy, and while specific questions in this field require continued study, the emerging evidence suggests surveillance for UGI neoplasia in Lynch syndrome is warranted and should be considered as part of a Lynch syndrome cancer risk management plan.

## Figures and Tables

**Table 1:** Summary of current guidelines addressing UGI cancer surveillance in Lynch syndrome.

Guideline	Year	Recommend Surveillance	Age to Initiate	Interval	*Helicobacter pylori* (Test and Treat)	Biopsy Recommendation	Quality of Evidence/Strength of Recommendation
European Society for Medical Oncology (ESMO) [16]	2013	Yes, for gastric cancer; Not for duodenal cancer	-	1–3 years, only in cases of individuals from “high incidence” populations	Yes	-	Not given
US Multi-Society Task Force [17]	2014	Yes	30–35	2–3 years “based on risk factors”	Yes	Gastric antrum	Expert consensus; GRADE rating: low
American College of Gastroenterology (ACG) [18]	2015	Yes	30–35	3–5 years, may be considered if there is a family history of gastric or duodenal cancer	Yes	Yes	Conditional recommendation, Very low quality of evidence
American Society of Clinical Oncology (ASCO) [15]	2015	Yes, for gastric cancer; Not for duodenal cancer	-	1–3 years in “in high incidence populations”	Yes	-	Not given
European Society of Digestive Oncology (ESDO) [19]	2018	Yes	No later than age 30	1–2 years	Yes	-	Not given
German Consortium for Familial Intestinal Cancer [20]	2019	Yes; for gastric and duodenal cancers	25	1–3 years	Yes	-	Not given
British Society of Gastroenterology (BSG) [14]	2020	No, only in context of clinical trial	-	-	Yes		Strong recommendation; GRADE rating: low
National Comprehensive Cancer Network (NCCN) [7]	2021	Consider	40	3–5 years in “high risk persons”: male, older age, *MLH1* or *MSH2* pathogenic variant, first degree relative with gastric cancer, Asian ethnicity, residing or immigrating from countries with high incidence of gastric cancer, chronic autoimmune gastritis, gastric intestinal metaplasia, gastric adenomas	Consider	Random biopsy of the proximal and distal stomach in high-risk persons	Category 2A: based upon lower-level evidence, there is uniform NCCN consensus that the intervention is appropriate
European Hereditary Tumour Group (EHTG) and European Society of Coloproctology (ESCP) [21]	2021	No	-	-	-	-	-

**Table 2:** Lifetime risk of gastric and small bowel cancers in Lynch syndrome by pathogenic variant [7,9,10,11,12].

Pathogenic Variant	Affected Individuals, Lifetime Risk	General Population, Lifetime Risk

**Gastric Cancer**		
***MLH1***	5–7%	0.9%
***MSH2*** **and *EPCAM***	0.2–9%	
***MSH6***	≤1–7.9%	
***PMS2***	Inadequate data	

**Small Bowel Cancer**		
***MLH1***	0.4–11%	0.3%
***MSH2*** **and *EPCAM***	1.1–10%	
***MSH6***	≤1–4%	
***PMS2***	0.1–0.3%	

**Table 3:** Studies evaluating surveillance of UGI cancer in Lynch syndrome.

Author Year Country	Study Design	Pre-Cancerous Lesion Detection	Cancers and Staging Detection	Conclusions
**Kumar** [32] **2020** **United States**	Retrospective, Registry Indication for EGD: symptoms or surveillance N = 217	◦ BE: 3.2% ◦ GIM: 8.3% ◦ *H pylori*: 2.8% ◦ Duodenal adenomas: 1.8%	◦ UGI cancer: 5% (11/217) ◦ 1 esophageal squamous cell, 6 gastric adenocarcinomas, 4 duodenal adenocarcinomas ◦ 5/11 cancers detected on surveillance, 80% stage I ◦ 6/11 detected on diagnostic EGD, 33% stage I	◦ EGD surveillance associated with: ◦ Early-stage cancer detection
**Farha** [31] **2021** **United States**	Retrospective, Registry Indication for EGD: asymptomatic surveillance N = 323	◦ BE: 6.5% ◦ *H pylori*: 3.8% ◦ GIM: 5.7% ◦ Gastric adenomas: 0.6% ◦ Gastric hyperplastic polyps > 5mm: 1.9% ◦ Duodenal adenomas: 1.5%	◦ UGI cancer: 1.5% (5/323) ◦ 1 esophageal adenocarcinoma, 1 gastric adenocarcinoma, 1 gastric NET, 2 duodenal adenocarcinomas ◦ 4/5 (80%) detected at stage I and 1 patient at stage IIB	◦ EGD surveillance associated with: ◦ Early-stage cancer detection ◦ Clinically actionable findings on both baseline and surveillance EGD
				
**Ceravolo** [45] **2021** **United States**	Retrospective Indication for EGD: asymptomatic surveillance N = 247	◦ H pylori: 6.9% ◦ GIM: 10.1% ◦ Gastric adenoma: 0.8% ◦ Duodenal adenoma: 2.8% ◦ Ampullary adenoma: 0.8%	◦ Gastric cancer: 0.8% (2/247); one stage pT1a and one stage pT3 ◦ Duodenal cancer: 0.8% (2/247), one stage pT2 and one stage T1	◦ EGD surveillance is useful to: ◦ Detect precancerous and cancerous UGI lesions
**Vangala** [65] **2021** **Germany**	Retrospective, Registry Indication for EGD: symptoms or surveillance N = 2015	◦ None described	◦ Duodenal cancers in surveillance group: 13/27 (48.1%); 77% early stage (I–IIA) ◦ Duodenal cancers in diagnostic group: 14/27 (51.9%); 29% early stage (I–IIA)	◦ EGD surveillance associated with: ◦ Early detection of duodenal cancers
**Galiatsatos** [72] **2017** **Turkey**	Retrospective Indication for EGD: not specified N = 21	◦ H pylori: 9.5% ◦ Atrophic gastritis: 0% ◦ GIM: 9.5%	◦ No cancers identified	◦ EGD surveillance not associated with: ◦ Detection of upper gastrointestinal cancers
**Renkonen-Sinisalo** [73] **2002** **Finland**	Prospective one-time EGD, case–control study including gastric biopsy N = 73 with Lynch syndrome and 32 mutation-negative family members	◦ Case/Control ◦ *H pylori:* 26/28% ◦ Atrophic gastritis: 14/22% ◦ GIM: 14/19%	◦ Duodenal cancer: 1.4% (1/105)	◦ EGD surveillance not associated with: ◦ Detection of early stage cancer ◦ Detection of premalignant lesions
**Ladigan-Badura S** [46] **2021** **Germany**	Review of gastric cancer cases in the German Consortium for Familial Intestinal Cancer Registry Indication for EGD: symptoms or surveillance N = 1128	◦ None described	◦ Gastric cancer: 2.3% (47/2009) ◦ 6/22 patients had cancer detected on surveillance; 83% Stage I ◦ 16/22 patients with no surveillance had cancer diagnosed; 25% Stage I	◦ Surveillance EGD associated with: ◦ Early-stage cancer detection
**Hammoudi** [62] **2019** **France**	Retrospective, Assessment of duodenal neoplasia on EGD or push EGD Indication for EGD: not specified N = 154	◦ Duodenal adenomas: 1.9%	◦ Duodenal cancer: 2.6% (4/154) ◦ 75% of patients with duodenal cancer diagnosed at advanced stage	◦ Surveillance EGD associated with: ◦ Detection of pre-cancerous and cancerous duodenal lesions

BE: Barrett’s esophagus; GIM: gastric intestinal metaplasia; NET: neuroendocrine tumor; EGD: Esophagogastroduodenoscopy.
